# The Role of Health Care Experience and Consumer Information Efficacy in Shaping Privacy and Security Perceptions of Medical Records: National Consumer Survey Results

**DOI:** 10.2196/medinform.3238

**Published:** 2015-04-02

**Authors:** Vaishali Patel, Ellen Beckjord, Richard P Moser, Penelope Hughes, Bradford W Hesse

**Affiliations:** ^1^U.S. Department of Health and Human ServicesOffice of the National Coordinator for Health Information TechnologyWashington, DCUnited States; ^2^University of PittsburghDepartment of PsychiatryPittsburgh, PAUnited States; ^3^National Institutes of HealthNational Cancer InstituteScience of Research and Technology BranchBethesda, MDUnited States; ^4^National Institutes of HealthNational Cancer InstituteHealth Communication and Informatics Research BranchBethesda, MDUnited States

**Keywords:** electronic health records, privacy confidentiality, health information exchange, health surveys, health knowledge, attitudes, practice

## Abstract

**Background:**

Providers’ adoption of electronic health records (EHRs) is increasing and consumers have expressed concerns about the potential effects of EHRs on privacy and security. Yet, we lack a comprehensive understanding regarding factors that affect individuals’ perceptions regarding the privacy and security of their medical information.

**Objective:**

The aim of this study was to describe national perceptions regarding the privacy and security of medical records and identify a comprehensive set of factors associated with these perceptions.

**Methods:**

Using a nationally representative 2011-2012 survey, we reported on adults’ perceptions regarding privacy and security of medical records and sharing of health information between providers, and whether adults withheld information from a health care provider due to privacy or security concerns. We used multivariable models to examine the association between these outcomes and sociodemographic characteristics, health and health care experience, information efficacy, and technology-related variables.

**Results:**

Approximately one-quarter of American adults (weighted n=235,217,323; unweighted n=3959) indicated they were very confident (n=989) and approximately half indicated they were somewhat confident (n=1597) in the privacy of their medical records; we found similar results regarding adults’ confidence in the security of medical records (very confident: n=828; somewhat confident: n=1742). In all, 12.33% (520/3904) withheld information from a health care provider and 59.06% (2100/3459) expressed concerns about the security of both faxed and electronic health information. Adjusting for other characteristics, adults who reported higher quality of care had significantly greater confidence in the privacy and security of their medical records and were less likely to withhold information from their health care provider due to privacy or security concerns. Adults with higher information efficacy had significantly greater confidence in the privacy and security of medical records and less concern about sharing of health information by both fax and electronic means. Individuals’ perceptions of whether their providers use an EHR was not associated with any privacy or security outcomes.

**Conclusions:**

Although most adults are confident in the privacy and security of their medical records, many express concerns regarding sharing of information between providers; a minority report withholding information from their providers due to privacy and security concerns. Whether individuals thought their provider was using an EHR was not associated with negative privacy/security perceptions or withholding, suggesting the transition to EHRs is not associated with negative perceptions regarding the privacy and security of medical information. However, monitoring to see how this evolves will be important. Given that positive health care experiences and higher information efficacy were associated with more favorable perceptions of privacy and security, efforts should continue to encourage providers to secure medical records, provide patients with a “meaningful choice” in how their data are shared, and enable individuals to access information they need to manage their care.

## Introduction

The Health Information Technology (HITECH) Act of 2009 put a number of federally funded initiatives in place to support the adoption and “meaningful use” of electronic health records (EHRs) by eligible providers, including physicians and hospitals [1,2]. These included financial incentives, initiatives to develop standards to exchange information electronically, and technical support for providers to adopt and use EHRs. A key and important element, which the HITECH Act also emphasized, was the importance of ensuring patient and provider trust in EHRs and the electronic exchange of health information [3].

The evolution of both technology and policy to address privacy and security needs is critical as providers’ use of EHRs has grown rapidly since HITECH [4,5]. As of 2012, almost three-quarters of physicians reported adopting an EHR [6]. As EHRs become the norm, a majority of patients’ medical records will become digitized, enabling providers to share health information electronically with other providers to better coordinate care.

As we transition from a paper-based to an electronic system of storing and sharing medical records and we make advancements to ensure the privacy and security of electronic health information, it is critically important to understand how consumers perceive these developments. Consumers represent important stakeholders in this process because it is their health information that is being digitized and shared electronically.

Consumers have expressed a desire for greater transparency and control over their health information, which many see as a key aspect of ensuring privacy [7]. Ensuring safeguards are in place to protect medical records so the information remains confidential is also an important concern and is considered a fundamental component of security [7]. A number of studies have suggested that consumer perspectives regarding the privacy and security of electronic health information are complex and varied. National surveys have shown that there is widespread concern about the privacy and security of EHRs and electronic health information exchange (HIE), with about half of individuals reporting in a recent survey that they expect EHRs to worsen privacy and security [7,8]. However, other findings indicate that many view EHRs as enhancing certain elements of privacy such as providing patients with greater control over their information and transparency regarding who accesses their information [9]. Findings across several surveys also suggest that a majority of individuals understand the potential benefits of EHRs and HIE [7-9], and consider these benefits to outweigh the potential privacy risks [7,10,11].

Evidence is still emerging regarding how these complex perspectives and growing adoption of EHRs may affect consumers’ perceptions regarding the privacy and security of their own medical records, including the sharing of their data among providers and patient-provider communication. Few studies have examined the association between consumer privacy and security concerns with provider EHR adoption [8].

With the increasing adoption of health information technology (IT), the Office of the National Coordinator for Health IT seeks to monitor general trends as well as identify key factors associated with individuals’ perceptions of privacy and security of medical information. Using data from a nationally representative survey of adults conducted by the National Cancer Institute (NCI) in 2011-2012, we sought to answer the following questions:

1. How confident are adults in the privacy and security of their medical records? What technology-related care experience and patient engagement–related factors are associated with consumer confidence in privacy and security?
2. What proportion of adults have withheld information from their provider due to privacy or security concerns? What technology-related care experience and information efficacy–related factors are associated with withholding information?
3. What are adults’ levels of concern regarding sending health medical information from one provider to another? Does this vary by whether it is sent by fax or electronically, and what differentiates adults who express concerns about these different modes of sharing electronic health information?

## Methods

### Data Collection and Response Rates

The data presented here are from the 2011-2012 administration of the NCI Health Information National Trends Survey (HINTS). HINTS is a nationally representative survey of the US noninstitutionalized adult population (≥18 years) that tracks attitudes, knowledge, and behavior related to health and cancer communication and health outcomes, with an emphasis on the evolution of health information technology in health care [12,13]. Data collection for the fourth iteration of HINTS (HINTS 4 Cycle 1) began in October 2011 and concluded in February of 2012 (N=3959), and included new items related to privacy and security of medical information. There are 3 more cycles of data collection planned through 2014. Data were collected via a self-administered mailed questionnaire using a comprehensive national listing of household addresses available from the United States Postal Service using a 2-stage, stratified sample. Within households, respondents were chosen using a randomized selection process. The final response rate for the postal survey was 36.7%, which is congruent with norms for federally funded population surveys. Full-sample and replicate weights were computed and are available to obtain population-level estimates and correct variance estimates, respectively. These weights correct for nonresponse and noncoverage to the extent possible. In creating these weights, sampling errors are reduced through the use of calibration variables from the American Community Survey (ACS) of the US Census Bureau based on the following demographic variables: age, gender, education, marital status, race, ethnicity, and census region. In addition, 2 other calibration variables from the National Health Interview Survey (NHIS) were used; namely, health insurance status and cancer status. Thus, weighted estimates of these calibration variables using the HINTS data will agree with those from the source data. Full details on the survey design and sampling strategies for the HINTS program have been published elsewhere [12,14].

### Outcome Measures

The NCI and the ONC worked collaboratively to create new HINTS items to assess perceptions about privacy and security of medical information. These questions underwent multiple rounds of cognitive testing to assess their validity using respondents who represented a range of levels of education, age, and health status.

The definitions for the items related to security and privacy were developed from the National Committee on Vital and Health Statistics (NCVHS) [15]. According to NCVHS, health information privacy is an individual’s right to control the acquisition, uses, or disclosures of his or her identifiable health data. Security refers to physical, technological, or administrative safeguards or tools used to protect identifiable health data from unwarranted access or disclosure.

Security concerns were assessed with the question “How confident are you that safeguards (including the use of technology) are in place to protect your medical records from being seen by people who aren’t permitted to see them?” Response options included very confident, somewhat confident, and not confident.

Privacy concerns were assessed with the question “How confident are you that you have some say in who is allowed to collect, use, and share your medical information?” Response options included very confident, somewhat confident, and not at all confident.

Withholding of information was assessed by asking: “Have you ever kept information from your health care provider because you were concerned about the privacy or security of your medical record?” (yes/no).

Finally, concerns regarding sending medical information to providers were assessed through 2 questions. The first was in regards to sending information by fax and asked respondents: “If your medical information is sent by fax from one health care provider to another, how concerned are you that an unauthorized person would see it?” (very concerned, somewhat concerned, not at all concerned). The second question focused on medical information “sent electronically from one health care provider to another” with the same response options.

### Independent Variables and Measures

#### Overview

The complex perspectives regarding privacy and security of medical and health information suggest a variety of factors may be involved that go beyond sociodemographic and health-related factors, which have been the focus of some studies [7,16]. Experience with technology, including their providers’ use of an EHR, may affect how individuals perceive privacy and security of their medical information. Additionally, individuals’ experiences with their health care provider may affect their level of confidence in the ability of the provider to maintain the privacy and security of their medical records and their subsequent withholding of information from their health care provider. How individuals seek out and obtain health information may affect their perceptions related to privacy and security of their medical information. Individuals with greater health information efficacy are more likely to seek out health information and make health care decisions on their own, and have higher levels of health literacy and numeracy [17-19]. Individuals in poor health may also perceive privacy and security of their medical records differently than those who are healthy and have fewer encounters with the health care system. Thus, in addition to sociodemographic characteristics (age, education, race/ethnicity, and gender) and health status, we also included the following variables in multivariate analyses.

#### Health Care Experience-Related Variables

Respondents reported on the quality of care received in the past 12 months from their health care provider (excellent, very good, good, fair/poor, no health care visits in the past 12 months) and their trust in information provided by a health care provider (a lot, some, a little/not at all).

#### Technology-Related Variables

An index of Internet activity was created to assess the degree to which respondents were engaged in online behaviors. This index considered use of the Internet, use of a personal health record (PHR), use of email to communicate with a health care provider, and having downloaded health information from the Internet. Respondents were given a score of “0″ if they did not use the Internet, “1″ if they used the Internet but did not engage in any of the 3 specific activities included in the index, “2″ if they used the Internet and had engaged in 1 of the 3 activities, and “3″ if they used the Internet and had engaged in 2 or 3 of the 3 activities. Participants were also asked “As far as you know, do your health care providers maintain your medical records in a computerized system?” (we use the term electronic “health” record although the survey items use the term “medical records”). The survey sought to ask about perceptions neutral of whether it was a paper-based system or electronic system.

#### Information Efficacy

Information efficacy was assessed with the question “Overall, how confident are you that you could get advice or information about health or medical topics if you needed it?” (completely confident, very confident, somewhat/a little/not at all confident).

### Data Analyses

We used SUDAAN version 10.01 [20] to account for the complex sampling procedure used by HINTS and to incorporate the final sample and jackknife replicate weights needed to produce nationally representative point estimates and correct standard errors, respectively. Descriptive statistics were used to provide population-level estimates for American adults’ (1) perceptions of privacy and security of medical records, (2) choice to withhold medication information from health care providers because of privacy or security concerns, and (3) relative concerns about the transmission of medical information via electronic means or via fax. Bivariate analyses estimated the degree to which privacy and security concerns were related, and how each of these was associated with choosing to withhold medical information. Finally, multinomial generalized logit models were used to estimate the relative odds of having concerns about security, privacy, or unauthorized access to faxed versus electronically transmitted health information. Multivariable logistic regression was used to estimate the odds of withholding information from a health care provider due to concerns about privacy and/or security. Predicted probabilities were also computed.

## Results

### Respondent Characteristics

Table 1 shows the sociodemographic characteristics of the nationally representative sample. In all, 58.58% (2443/3924) of adults reported that over the past 12 months they had received either excellent (28.73%, 1190/3924) or very good quality of care (29.85%, 1253/3924). A quarter of adults reported they were completely confident in their ability to obtain health-related advice or information they needed (25.53%, 1002/3931), whereas more than one-third (38.81%, 1531/3931) reported they were only somewhat, a little, or not at all confident in their abilities to do so. With regards to technology exposure and uptake, approximately one-quarter (26.26%, 957/3621) of adults engaged in some health-related activity online. The majority (83.99%, 3332/3855) reported their providers kept their medical records in a computerized format.

**Table 1:** Respondent characteristics (N=3959).

Variable		Unweighted sample size, n	Respondents, %
Age (years), weighted mean (SD)		3891	46.47 (0.09)
**Education**			
	Less than high school	391	12.9
	High school	785	23.1
	Some college	1167	31.1
	College grad or more	1531	32.9
**Race/ethnicity**			
	Hispanic	461	14.5
	Non-Hispanic white	2431	66.8
	Non-Hispanic African American	576	11.4
	Non-Hispanic Asian	168	5.0
	Other/multiple	103	2.4
**Gender**			
	Female	2304	51.5
**Health status**			
	Excellent	496	13.6
	Very good	1398	37.1
	Good	1397	34.2
	Fair/poor	632	15.1
**Overall quality of care in the past 12 months**			
	Excellent	1190	28.7
	Very good	1253	29.9
	Good	590	13.7
	Fair/poor	254	6.6
	No visits in the last 12 months	637	21.1
**How much do you trust information from a health care provider?**			
	A lot	2685	71.0
	Some	1001	23.1
	A little/not at all	230	6.0
**Internet activity index**			
	Not online	1043	24.7
	Online, but does not use a PHR, email providers, or download health information	1621	49.1
	Online and does 1 of the 3 activities	588	16.3
	Online and does 2 or 3 of the 3 activities	369	10.0
**How confident are you that you could get health-related advice or information if you needed it?, n (%)**			
	Completely	1002	25.5
	Very	1398	35.7
	Somewhat/a little/not at all	1531	38.8
**As far as you know, does your health care provider keep your medical records in a computerized format?**			
	Yes	3332	84.0

#### Confidence Regarding Privacy and Security of Medical Information

Overall, three-quarters of adults reported they were very or somewhat confident in the security of their medical records (75.45%, 2570/3461). Three-quarters of adults also reported they were either very or somewhat confident in the privacy of their medical records (75.41%, 2586/3469).

**Figure 1:**
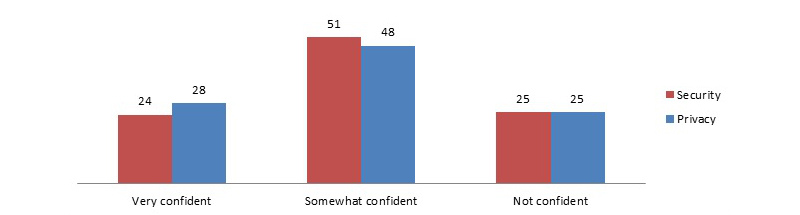
National perceptions regarding confidence in the privacy and security of medical records (data source: HINTS 4 Cycle 1, 2011-2012).

The distribution of adults’ level of confidence regarding the privacy and security of their medical records was fairly similar (Figure 1). Concerns about security and privacy were related: among those who were very confident in the security of their medical records, 75.1% (644/826) were also very confident in the privacy of their medical records (χ^2^_4_=99.9, *P*<.001).

#### Factors Associated With Confidence in Privacy and Security

**Figure 2:**
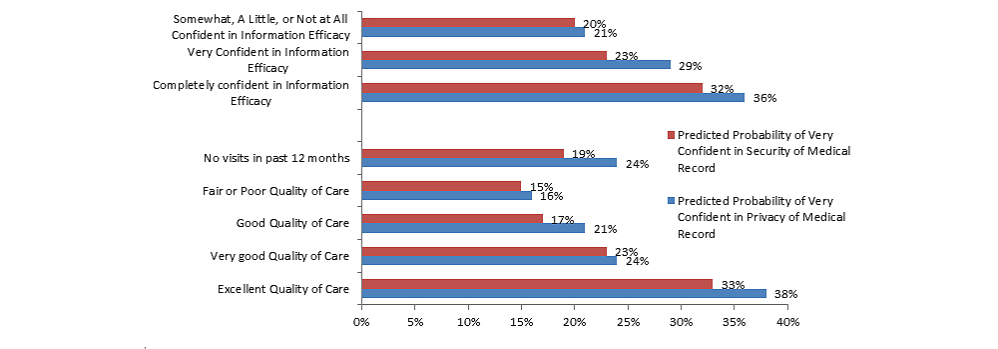
Perceptions regarding privacy and security of medical records by quality of care received and information efficacy (data source: HINTS 4 Cycle 1, 2011-2012).

In multivariate analyses, reported quality of care and information efficacy were significantly associated with perceptions of privacy and security (Figure 2). Specifically, adjusting for other characteristics, predicted probabilities estimated from the model indicated that more than twice as many adults receiving high quality of care reported being very confident in the privacy of their medical information as compared to those who received fair or poor quality of care (38.11% vs 15.69%, *P*<.001). Similarly, twice as many adults who received high-quality care reported they were very confident in the security of their medical records (33.19% vs 14.51%, *P*<.001). Approximately one-third of adults with higher levels of information efficacy reported they were very confident in the privacy of their medical information (35.92%) or the security of their medical information (31.79%) compared to approximately one-fifth of adults with low levels of efficacy (both *P*<.001).

Additionally, Hispanics, African-Americans (*P*=.03 for privacy, *P*<.001 for security), and women had significantly (both *P*<.001) higher odds of reporting greater confidence in the privacy and security of their medical information (Table 2). Provider EHR use was not associated with confidence in privacy or security of medical records.

**Table 2:** Multivariate models of concerns about security and privacy.

Variable		Privacy, AOR (95% CI)			Security, AOR (95% CI)		
		Very confident vs not confident	Somewhat confident vs not confident	*P*	Very confident vs not confident	Somewhat confident vs not confident	*P*
Age		0.99 (0.97, 1.00)	0.99 (0.98, 1.00)	.64	0.99 (0.98, 1.01)	1.00 (0.98, 1.01)	.06
**Education**							
	Less than high school	4.90 (2.17, 11.06)	1.67 (0.89, 3.11)		2.37 (0.98, 5.71)	1.14 (0.45, 2.86)	
	High school	2.41 (1.45, 3.98)	1.24 (0.83, 1.83)		1.61 (0.96, 2.69	1.31 (0.82, 2.09)	
	Some college	1.44 (0.96, 2.15)	1.28 (0.90, 1.81)		1.27 (0.79, 2.02)	1.22 (0.78, 1.91)	
	College grad or more	Reference	Reference	.39	Reference	Reference	.01
**Race/ethnicity**							
	Hispanic	3.06 (1.60, 5.87)	1.66 (0.89, 3.13)		1.91 (1.10, 3.30)	1.28 (0.71, 2.31)	
	Non-Hispanic white	Reference	Reference	.03	Reference	Reference	<.001
	African American	2.78 (1.83, 4.23)	2.40 (1.45, 3.99)		2.55 (1.44, 4.52)	1.51 (0.80, 2.84)	
	Asian	1.72 (0.75, 3.95)	1.16 (0.52, 2.61)		1.05 (0.52, 2.11)	0.82 (0.32, 2.12)	
	Other/multiple	1.92 (0.79, 4.70)	1.53 (0.79, 2.97)		1.66 (0.62, 4.38)	1.77 (0.86, 3.64)	
**Gender**							
	Female	1.70 (1.30, 2.21)	1.23 (0.95, 1.60)	<.001	2.22 (1.68, 2.95)	1.27 (0.95, 1.68)	<.001
**Health status**							
	Excellent	Reference	Reference	.13	Reference	Reference	.01
	Very good	0.92 (0.59, 1.42)	0.99 (0.60, 1.65)		1.27 (0.63, 2.57)	1.40 (0.71, 2.79)	
	Good	1.14 (0.70, 1.86)	0.99 (0.61, 1.62)		1.63 (0.90, 2.94)	1.58 (0.84, 2.98)	
	Fair/Poor	0.47 (0.26, 0.86)	0.71 (0.40, 1.27)		0.88 (0.44, 1.76)	1.31 (0.63, 2.71)	
**Overall quality of care**							
	Excellent	Reference	Reference	<.001	Reference	Reference	<.001
	Very good	0.57 (0.37, 0.89)	0.99 (0.65, 1.52)		0.36 (0.17, 0.78)	0.65 (0.34, 1.23)	
	Good	0.31 (0.16, 0.58)	0.72 (0.44, 1.19)		0.25 (0.13, 0.51)	0.55 (0.33, 0.92)	
	Fair/poor	0.17 (0.06, 0.43)	0.39 (0.19, 0.79)		0.16 (0.06, 0.43)	0.48 (0.24, 0.94)	
	No visits last 12 mo	0.28 (0.17, 0.47)	0.53 (0.32, 0.89)		0.32 (0.18, 0.58)	0.57 (0.33, 0.99)	
**Trust in HCP**							
	A lot	Reference	Reference	.23	Reference	Reference	<.001
	Some	0.42 (0.27, 0.67)	0.54 (0.40, 0.73)		0.58 (0.36, 0.95)	0.77 (0.53, 1.12)	
	A little/Not at all	0.61 (0.29, 1.27)	0.50 (0.25, 1.00)		0.71 (0.36, 1.40)	0.82 (0.45, 1.49)	
**Internet activity index**							
	Not online	1.03 (0.61, 1.75)	1.03 (0.67, 1.57)		1.47 (0.82, 2.61)	0.89 (0.52, 1.52)	
	Online but no health activity online	Reference	Reference	.08	Reference	Reference	.87
	Online and does 1 of 3 health activities (PHR, email doctor, download health information)	0.88 (0.58, 1.32)	0.76 (0.51, 1.14)		1.19 (0.75, 1.89)	0.75 (0.51, 1.12)	
	Online and does 2 or 3 health activities online	0.93 (0.54, 1.60)	0.95 (0.65, 1.38)		1.02 (0.52, 1.99)	0.86 (0.52, 1.43)	
**Information efficacy**							
	Completely	Reference	Reference	<.001	Reference	Reference	<.001
	Very	0.67 (0.49, 0.92)	1.10 (0.72, 1.68)		0.83 (0.51, 1.33)	1.27 (0.79, 2.06)	
	Somewhat/a little/not at all	0.52 (0.36, 0.74)	1.08 (0.78, 1.49)		0.36 (0.21, 0.62)	0.78 (0.51, 1.18)	
**Provider EHR use**							
	Yes	Reference	Reference	.99	Reference	Reference	.59
	No	0.80 (0.49, 1.32)	0.84 (0.56, 1.28)		1.00 (0.55, 1.81)	1.03 (0.61, 1.73)	

#### Withholding of Information Because of Privacy or Security Concerns

**Figure 3:**
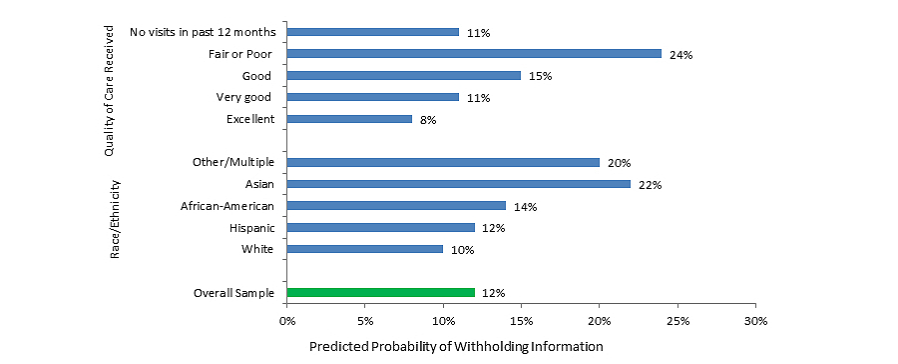
Predicted probability of withholding of information from health care providers due to privacy or security concerns (data source: HINTS 4 Cycle 1, 2011-2012).

A total of 12.33% (520/3904) of adults reported they had kept information from their health care provider because of concerns about the privacy and security of their medical information (Figure 3).

#### Factors Related to Withholding Information From a Health Care Provider

As shown in Figure 3, adjusting for other characteristics, individuals who rated the quality of care they received as lower (fair or poor) had 3 times the predicted probability of withholding information compared to those who received excellent care (23.93% vs 8.39%, *P*=.02). Asian-Americans had 2 times higher predicted probability of withholding information from their health care provider due to privacy or security concerns compared to white non-Hispanics (22.39% vs 9.90%, *P*=.01). Provider EHR use was not associated with withholding due to privacy or security concerns (Table 3).

**Table 3:** Multivariate model regarding withholding of health information from health care provider due to privacy or security concerns.

Variable		Ever withheld health information (yes vs no), AOR (95% CI)	*P*
Age		1.00 (0.99, 1.01)	.70
**Education**			
	Less than high school	0.87 (0.39, 1.96)	
	High school	0.67 (0.36, 1.23)	
	Some college	1.19 (0.82, 1.72)	
	College grad or more	Reference	.24
**Race/ethnicity**			
	Hispanic	1.31 (0.78, 2.21)	
	Non-Hispanic white	Reference	.01
	African American	1.56 (0.84, 2.90)	
	Asian	2.73 (1.43, 5.22)	
	Other/multiple	2.29 (0.82, 6.41)	
**Gender**			
	Female	1.30 (0.90, 1.87)	.15
**Health status**			
	Excellent	Reference	.80
	Very good	1.22 (0.77, 1.93)	
	Good	1.06 (0.67, 1.70)	
	Fair/poor	1.18 (0.51, 2.72)	
**Overall quality of care**			
	Excellent	Reference	.02
	Very good	1.40 (0.90, 2.16)	
	Good	1.96 (1.13, 3.42)	
	Fair/poor	3.57 (1.64, 7.75)	
	No visits last 12 mo	1.33 (0.72, 2.47)	
**Trust in HCP**			
	A lot	Reference	.23
	Some	1.35 (0.87, 2.10)	
	A little/not at all	0.74 (0.36, 1.52)	
**Internet activity index**			
	Not online	0.91 (0.45, 1.84)	
	Online but no health activity online	Reference	.06
	Online and does 1 of 3 health activities (PHR, email doctor, download health information)	0.79 (0.49, 1.28)	
	Online and does 2 or 3 health activities online	1.57 (0.98, 2.51)	
**Information efficacy**			
	Completely	Reference	.92
	Very	1.08 (0.71, 1.66)	
	Somewhat/a little/not at all	1.09 (0.69, 1.71)	
**Provider EHR use**			
	Yes	Reference	.78
	No	0.93 (0.55, 1.56)	

#### Concerns Regarding Sending Medical Information Between Providers

A majority of individuals expressed they were either very or somewhat concerned about unauthorized individuals viewing their data when it is sent between health care providers, whether by fax or electronic means (data not shown). A quarter of adults were “very concerned” (24.89%, 892/3474) about unauthorized persons gaining access to faxed health information compared to 18.75% (724/3462) if the information was sent electronically. Similar proportions of individuals expressed they were “somewhat concerned” about fax (42.12%, 1476/3474) or electronically (45.77%, 1566/3462) sending of information. Approximately one-third of individuals expressed they were not concerned about fax (32.99%, 1106/3474) or electronic (35.47%, 1172/3462) means of transmitting their health information between providers.

**Figure 4:**
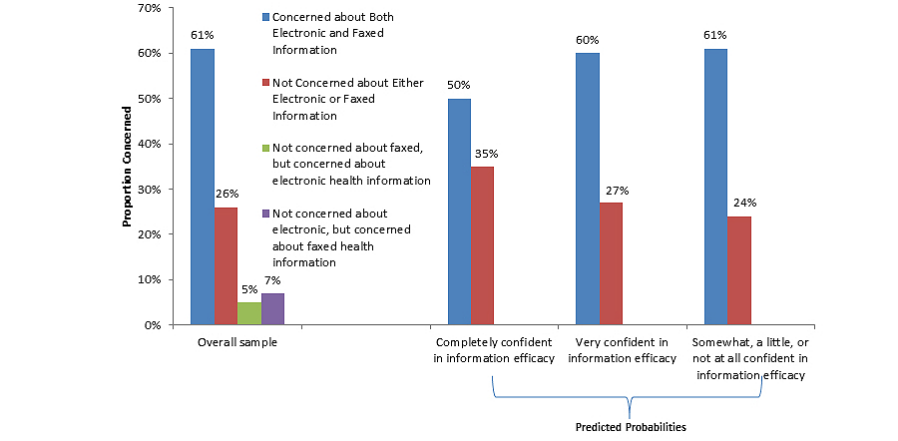
National concerns about exchanging medical information between providers by methods of exchange and information efficacy (data source: HINTS 4 Cycle 1, 2011-2012).

When responses to these questions were combined to understand the percentage of adults who were concerned about both methods of transmission, neither or only 1 or the other, a majority of adults (59.06%, 2100/3459) indicated they were either very or somewhat concerned about both electronically sending or faxing their health information, whereas approximately one-quarter (27.55%, 914/3459) were not concerned about either method. Very small proportions of adults were concerned about electronically exchanging data only (5.44%, 188/3459) or faxing data only (7.94%, 257/3459) (Figure 4).

#### Factors Associated with Concerns Regarding Methods of Sharing Data Between Providers

As shown in Figure 4, adults with higher levels of information efficacy had a significantly lower predicted probability of being concerned about both fax and electronic means of sending information between providers compared to adults with lower levels of information efficacy (49.61% vs 60.28%-61.15%, *P*=.02). Provider EHR use was not associated with concerns regarding methods of sharing data between providers (Table 4).

**Table 4:** Multivariate models of concerns about faxed versus electronic health information.

Variable		Concerned about both vs not concerned about either, OR (95% CI)	Concerned about fax vs not concerned about either, OR (95% CI)	Concerned about electronic vs not concerned about either, OR (95% CI)	*P*
**Age**		1.00 (0.99, 1.01)	0.99 (0.98, 1.01)	1.00 (0.98, 1.02)	.68
**Education**					
	Less than high school	0.75 (0.37, 1.53)	0.97 (0.27, 3.47)	1.55 (0.22, 11.04)	
	High school	1.05 (0.71, 1.55)	1.22 (0.53, 2.81)	0.51 (0.23, 1.13)	
	Some college	1.26 (0.93, 1.70)	0.85 (0.53, 1.35)	0.85 (0.48, 1.50)	
	College grad or more	Reference	Reference	Reference	.16
**Race/ethnicity**					
	Hispanic	1.29 (0.75, 2.23)	1.07 (0.36, 3.21)	0.82 (0.25, 2.71)	
	Non-Hispanic white	Reference	Reference	Reference	.06
	African American	2.49 (1.46, 4.25)	1.50 (0.56, 4.04)	2.04 (0.27, 15.57)	
	Asian	2.86 (1.38, 5.95)	4.72 (1.20, 18.54)	2.59 (0.71, 9.43)	
	Other/multiple	0.99 (0.46, 2.13)	0.59 (0.14, 2.58)	0.63 (0.13, 3.15)	
**Gender**					
	Female	1.24 (0.94, 1.62)	1.36 (0.72, 2.57)	0.98 (0.59, 1.63)	.41
**Health status**					
	Excellent	Reference	Reference	Reference	.31
	Very good	1.23 (0.71, 2.13)	0.63 (0.31, 1.28)	1.07 (0.40, 2.86)	
	Good	1.08 (0.65, 1.77)	0.78 (0.43, 1.41)	0.69 (0.25, 1.85)	
	Fair/poor	1.26 (0.69, 2.29)	0.93 (0.32, 2.67)	0.99 (0.31, 3.16)	
**Overall quality of care**					
	Excellent	Reference	Reference	Reference	.09
	Very good	1.29 (0.80, 2.06)	1.00 (0.49, 2.05)	0.79 (0.41, 1.51)	
	Good	1.07 (0.68, 1.69)	0.36 (0.15, 0.84)	1.11 (0.51, 2.42)	
	Fair/poor	2.13 (0.96, 4.74)	2.58 (0.67, 9.93)	3.27 (0.49, 21.66)	
	No visits last 12 mo	1.19 (0.76, 1.85)	1.03 (0.45, 2.33)	1.80 (0.67, 4.86)	
**Trust in HCP**					
	A lot	Reference	Reference	Reference	.20
	Some	1.46 (0.96, 2.23)	1.02 (0.47, 2.20)	1.12 (0.51, 2.45)	
	A little/not at all	0.57 (0.25, 1.27)	0.24 (0.06, 0.90)	0.41 (0.13, 1.32)	
**Internet activity index**					
	Not online	1.51 (0.84, 2.73)	0.58 (0.22, 1.52)	1.25 (0.40, 3.92)	
	Online but no health activity online	Reference	Reference	Reference	.16
	Online and does 1 of 3 health activities (PHR, email doctor, download health information)	0.93 (0.64, 1.34)	1.50 (0.74, 3.07)	1.02 (0.45, 2.33)	
	Online and does 2 or 3 health activities online	1.44 (0.90, 2.30)	1.44 (0.82, 2.51)	1.16 (0.51, 2.64)	
**Information efficacy**					
	Completely	Reference	Reference	Reference	.05
	Very	1.61 (1.08, 2.40)	1.27 (0.66, 2.48)	0.86 (0.33, 2.23)	
	Somewhat/a little/not at all	1.82 (2.26, 2.63)	1.66 (0.88, 3.12)	1.08 (0.44, 2.67)	
**Provider EHR use**					
	Yes	Reference	Reference	Reference	.71
	No	1.26 (0.73, 2.17)	0.90 (0.41, 1.97)	0.93 (0.33, 2.60)	

## Discussion

As EHR adoption has increased dramatically, a majority of individuals report they are confident in the privacy and security of their medical records. Three-quarters of adults reported they were either very or somewhat confident in the security of their medical records; similarly, three-quarters of adults reported they were very or somewhat confident in the privacy of their medical records. However, we found evidence suggesting that concerns regarding privacy and security may have a negative influence on provider-patient interactions. In all, 12.33% of adults reported they have withheld information from their health care provider due to concerns about the privacy and security of their medical record. Additionally, a majority of adults (59.06%, 2100/3459) reported being very or somewhat concerned that an unauthorized person might view their medical information when it is sent between health care providers, regardless of whether the information was sent by fax or electronically.

Our findings suggest that, thus far, the transition from paper to electronic health records is not associated with negative perceptions regarding the privacy and security of individuals’ medical information. Individuals who believed their providers were using electronic modes of storing or sharing health information did not report lower levels of confidence or greater concerns. Our findings regarding the lack of association between perceptions of provider EHR adoption and privacy and security concerns are consistent with other national survey results, which also found a lack of association between whether an individuals’ doctor used an EHR and perceptions regarding the privacy of HIE [8]. Our findings also suggest that there is room for improvement because half of adults reported they were less than very confident in the privacy and security of their medical records; this is consistent with prior studies that have indicated a majority of individuals are concerned about the impact of EHRs on privacy and security [7,8]. Thus, monitoring this over time will be critical because these perceptions may evolve as exposure to providers’ use of health IT increases.

A majority of adults did express concerns about the sharing of information between health care providers, whether by fax or electronic means. Similar levels of individuals (between 64.52% and 67.01%) expressed being very or somewhat concerned about either fax or electronic means of sharing their data. Our findings suggest that safeguards and policies should focus on building trust in the exchange of health information between providers, regardless of method of exchange. Although relatively few had concerns specific to electronically sharing information only, ONC is leading several efforts to ensure that entities facilitating exchange implement appropriate privacy and security policies to protect the information as it flows electronically across organizations while also enabling patients to have a “meaningful choice” in how their information is exchanged [3]. For example, in order for providers to receive incentive payments, they must transmit health information using secure technology.

Lack of confidence in privacy and security of medical records seems to negatively affect patient-provider communication. Although a minority of individuals (12%) reported withholding information due to privacy and security concerns, the impact on their health care may be significant. A prior survey found that 4% of individuals reported they had avoided requesting medical care or filling a prescription due to concerns about privacy [21]. Another survey found that 15% of individuals reported they would withhold sensitive information if their providers could exchange health information electronically [22]. A recent study found that smokers were more likely to withhold information from their health care provider due to privacy and security concerns, suggesting that individuals with potentially stigmatizing health conditions may be more likely to withhold sensitive information [16]. Together, these findings suggest that greater privacy and security concerns may be associated with negative patient-provider interactions and that withholding may be specific to certain types of sensitive health information. There are federal initiatives underway that seek to segment or separate sensitive information from other types of electronically transmitted information to alleviate potential concerns [23]. Five pilot projects are underway to demonstrate the technical capability for exchanging sensitive health information so that a patient’s privacy preferences are honored.

We did find racial and ethnic differences in privacy and security perceptions as well as potential cultural differences affecting withholding of information due to privacy and security concerns. Our findings that African-Americans and Hispanic Americans had a higher likelihood of expressing they were “very confident” in the privacy and security of their medical records compared to white non-Hispanics does run counter to prior studies which have found individuals from racial/ethnic minorities expressing greater privacy and security concerns [7]. However, other studies also show high interest in health IT use and support for HIE among most minorities [24,25]. Thus, monitoring these perceptions over time to see if these patterns are anomalies or new patterns will be important to assess as future rounds of the HINTS survey are conducted. We also found that Asian-Americans were more likely to withhold health information. This finding is consistent with a smaller, community-based study that found a high proportion of Asian-Americans expressed lower levels of support for HIE and PHRs which may have been related to privacy or security concerns [24]. There will be an opportunity to monitor and validate our initial finding as a national survey on privacy and security funded by ONC in 2014 will be oversampling Asian-Americans. Other converging evidence is slated to be published from special emphasis studies using HINTS items in Guam and the People’s Republic of China [26].

Our findings also suggest that confidence in the security and privacy of medical records may be associated with perceptions of quality and a sense of engagement with the health care system. Adults who reported more positive appraisals of the quality of their health care tended to be the same ones who reported greater confidence in the privacy and security of their medical information and were less likely to withhold information from their health care provider due to privacy or security concerns. These findings underscore the important role that individuals perceive that providers play in maintaining privacy and security of medical records. Adults’ general confidence in the privacy and security of their medical records may be linked to high levels of trust in their health care provider to protect the privacy and confidentiality of their health information [9]. High-quality health care providers may also be perceived to be more rigorous in their maintenance of medical records. Future research should examine the adoption of privacy and security safeguards by providers.

Adults who reported a greater sense of information efficacy—that is, a sense of confidence in their ability to find and control the information they need for their own health and health care—also reported a greater sense of confidence in the privacy and security of their medical records and less concern about data transmitted between providers. Our findings suggest that if health information technology serves to empower individuals to successfully gain greater access and control over their health information, their positive perceptions regarding the privacy and security of their health information may increase. Providing individuals with greater access to their own health information and the ability to use that information to manage their health and health care of their loved ones is a central cornerstone of ONC’s strategy to advance the use of health IT to improve care [27]. Federal initiatives are trying to make this vision into a reality by increasing consumers’ access to their own health information through the incentive program requirements and through the Blue Button download initiative [28,29].

Our study assesses individuals’ perceptions using general definitions of privacy and security, but both these concepts consist of a variety of specific areas. For example, privacy includes openness, access, and use limitations, whereas security encompasses issues such as availability and integrity of information. This survey did not cover these individual domains and they warrant further investigation. Additionally, although these survey items were cognitively tested to ensure respondents’ understanding, it is possible that respondents had difficulty assessing the differences between privacy and security. These are self-reported data that cannot be verified. For example, respondent-reported rates of EHR adoption (86%) are higher than physician-reported rates (72%), suggesting that some individuals may have mistakenly thought their health care provider was using an EHR when they were using a practice management system. Although these individuals may have erroneously believed their provider was using an EHR, this study’s examination of the association between perceptions regarding individuals’ privacy and security of their medical records and their providers’ use of health information technology is valid given that the focus of this study on individuals’ perceptions. The response rate for the postal frame tended to be low, although it exceeds random digit dial surveys and is comparable to other federal surveillance mechanisms [30,31]. Efforts were made to address potential sources of error (eg, nonresponse) through poststratification weighting techniques [32].

This nationally representative survey provides timely data on individuals’ perceptions regarding privacy and security of their medical records and its association with health IT and care experiences. Although EHR adoption rates have increased, a majority of adults report they are very or somewhat confident in the privacy and security of their medical records. However, many individuals do express concerns regarding the sharing of medical information between providers. Furthermore, privacy and security concerns have led a small but significant minority of individuals to withhold information from their health care providers. Yet, we did not find an association between these concerns and negative impacts with EHR adoption or electronic HIE. Our findings suggest it will be important to continue monitoring the effects of EHR adoption and HIE on privacy and security attitudes and behaviors. Additionally, efforts should continue to encourage providers to secure medical records, provide patients with a meaningful choice in how their data are shared, and enable consumers to access information they need to manage their care.

## Abbreviations

ACS: American Community Survey
EHR: electronic health records
HIE: health information exchange
HINTS: Health Information National Trends Survey
HITECH: Health Information Technology
NCI: National Cancer Institute
NCVHS: National Committee on Vital and Health Statistics
NHIS: National Health Interview Survey
PHR: personal health record
